# Two new species of the genus *Opopaea* (Araneae, Oonopidae) from Myanmar

**DOI:** 10.3897/zookeys.917.48924

**Published:** 2020-03-09

**Authors:** Yanfeng Tong, Zengliang Chen, Shuqiang Li

**Affiliations:** 1 Life Science College, Shenyang Normal University, Shenyang 110034, China Shenyang Normal University Shenyang China; 2 Southeast Asia Biological Diversity Research Institute, Chinese Academy of Sciences, Yezin, Nay Pyi Taw 05282, Myanmar Southeast Asia Biological Diversity Research Institute, Chinese Academy of Sciences Yezin Myanmar; 3 The Sericultural Research Institute of Liaoning Province, Fengcheng, Liaoning 118100, China The Sericultural Research Institute of Liaoning Province Fengcheng China; 4 Institute of Zoology, Chinese Academy of Sciences, Beijing 100101, China Institute of Zoology, Chinese Academy of Sciences Beijing China

**Keywords:** Goblin spider, morphology, new species, taxonomy

## Abstract

Two new species of the genus *Opopaea* Simon, 1892 are reported from Myanmar, *O. kanpetlet* Tong & Li, **sp. nov.** (♂♀) and *O. zhigangi* Tong & Li, **sp. nov.** (♂♀). Morphological descriptions and photographic illustrations of the two new species are given. All types are preserved in the Institute of Zoology, Chinese Academy of Sciences in Beijing (IZCAS).

## Introduction

The genus *Opopaea* Simon, 1892 is a widespread and highly diverse goblin spider genus, with biodiversity hotspots in Africa, Asia and Australia (Baehr 2013). A total of 185 valid extant species are currently known, in which 46 in Africa, 33 in Asia, 96 in Australia and New Caledonia, and 10 in other areas (WSC 2020).

The genus *Opopaea* of Myanmar and the adjacent countries has been poorly studied. Brignoli (1978) reported a new species from Bhutan. Tong and Li (2013) reported two new species and one newly recorded species from Laos. There are no records of the genus *Opopaea* in Myanmar. However, four species of the genus *Gamasomorpha* Karsch, 1881 and two recently described species of the endemic genus *Kachinia* Tong & Li, 2018 have been reported from Myanmar (Tong and Li 2018; WSC 2020). The present paper expands the known oonopid diversity of Myanmar by adding one newly recorded genus and two new species.

## Materials and methods

The specimens were examined in 95% ethanol using a Leica M205C stereomicroscope. Details were studied with an Olympus BX51 compound microscope. Photos were taken with a Canon EOS 750D zoom digital camera (18 megapixels) mounted on an Olympus BX51 compound microscope. Scanning electron microscope images (SEM) were taken under high vacuum with a Hitachi TM3030 after critical point drying and gold-palladium coating. All measurements were taken using an Olympus BX51 compound microscope and are given in millimeters in the text. The materials are preserved in the Institute of Zoology, Chinese Academy of Sciences in Beijing (**IZCAS**).

Terminology mainly follows Andriamalala and Hormiga (2013). The following abbreviations are used in the text: AL = abdomen length; ALE = anterior lateral eyes; ALE-ALE = distance between ALE and ALE; ALE-PLE = distance between ALE and PLE; AW = abdomen width; CBL = cymbiobulbus length; CBW = cymbiobulbus width; CL = carapace length; CW = carapace width; EGW = eye group width; FI = femur insertion on patella; FML = femur length; PLE = posterior lateral eyes; PME = posterior median eyes; PME-PME = distance between PME and PME; PLE-PME = distance between PLE and PME; PTL = patella length; TL = total length. Used in the figures: apo = apodeme; asr = anterior scutal ridge; boc = booklung covers; cb = cymbiobulbus; dte = dorsolateral, triangular extensions; fm = femur; fn = fenestra; ga = globular appendix; nlp = nail-like process; pd = postgynal depression; pls = paddle-like sclerite; psr = posterior scutal ridge; pt = patella; sr = scutal ridge.

## Taxonomy

### Family Oonopidae Simon, 1890

#### Genus *Opopaea* Simon, 1892

##### Opopaea kanpetlet

Taxon classificationAnimaliaAraneaeOonopidae

Tong & Li sp. nov.

57DC4A9C-B4DD-5CDD-B759-74C76DF693F9

http://zoobank.org/DABDCD9E-6129-4704-A835-E64C97836930

**Figure 1.**
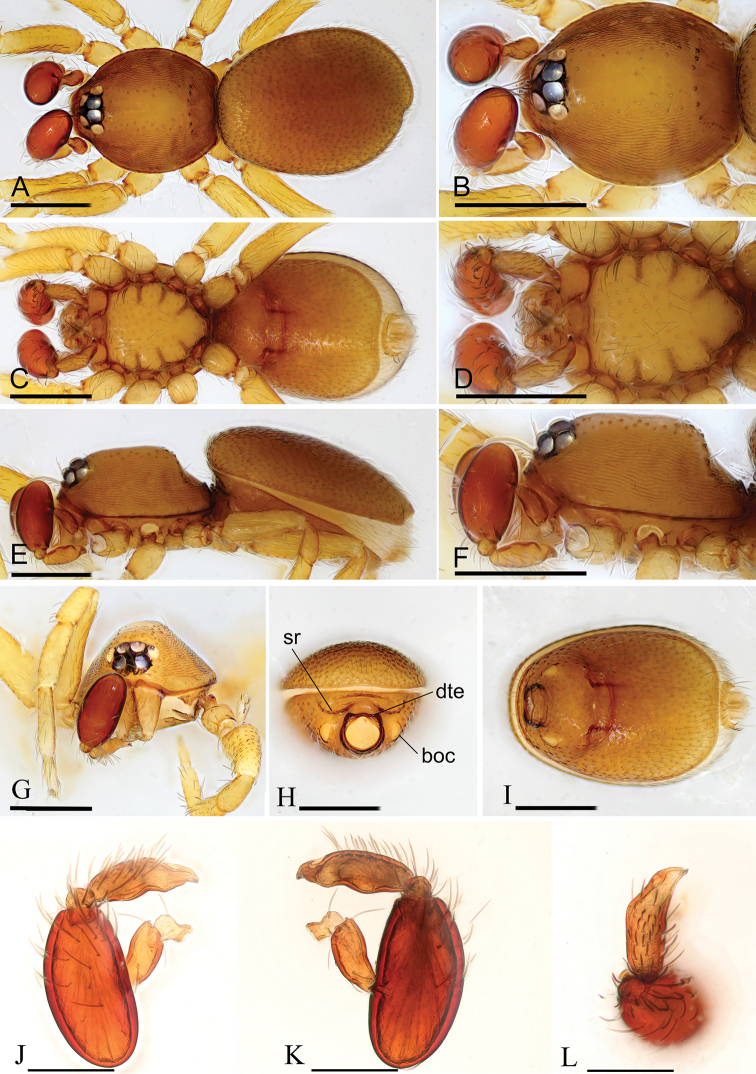
*Opopaea kanpetlet* sp. nov., male holotype **A, C, E** habitus, dorsal, ventral and lateral views **B, D, F, G** prosoma, dorsal, ventral, lateral and anterior views **H, I** abdomen, anterior and ventral views **J, K, L** left palp, prolateral, retrolateral and dorsal views. Abbreviations: boc = booklung covers; dte = dorsolateral, triangular extensions; sr = scutal ridge. Scale bars: 0.4 mm (**A–I**); 0.2 mm (**J–L**).

**Figure 2.**
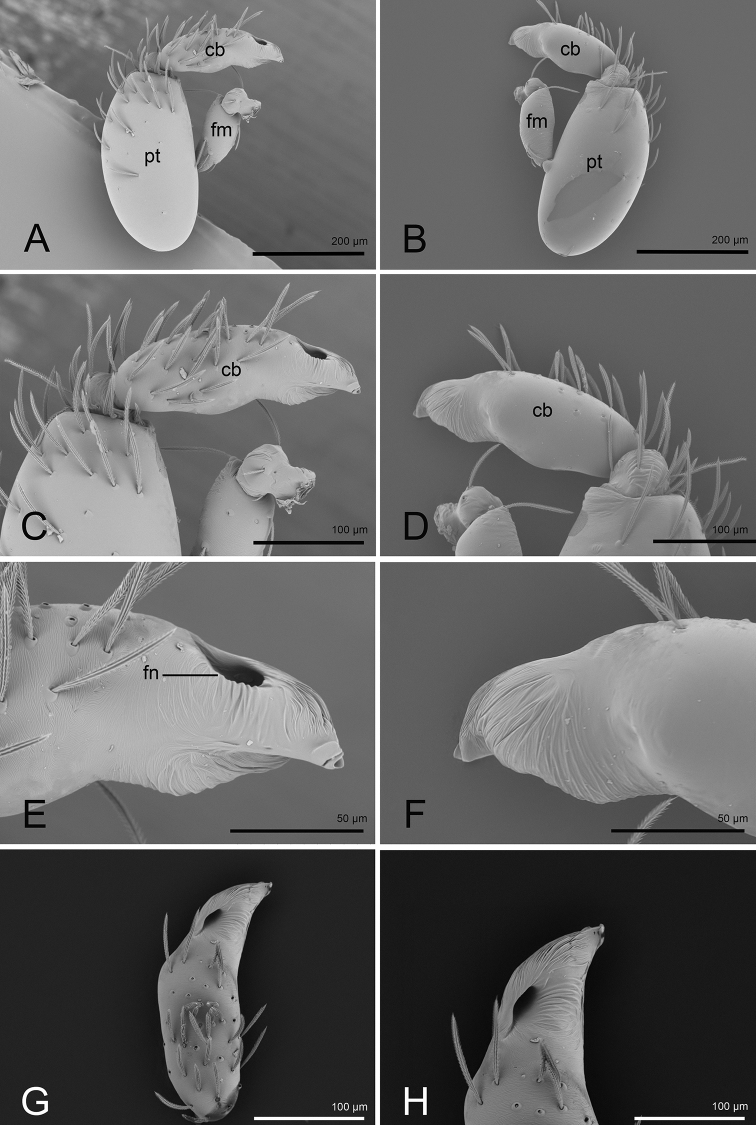
*Opopaea kanpetlet* sp. nov., holotype, left male palp, SEM**A, B** prolateral and retrolateral views **C, D, G** cymbiobulbus, prolateral, retrolateral and dorsal views **E, F, H** distal part of cymbiobulbus, prolateral, retrolateral and dorsal views. Abbreviations: cb = cymbiobulbus; fm = femur; fn = fenestra; pt = patella.

**Figure 3.**
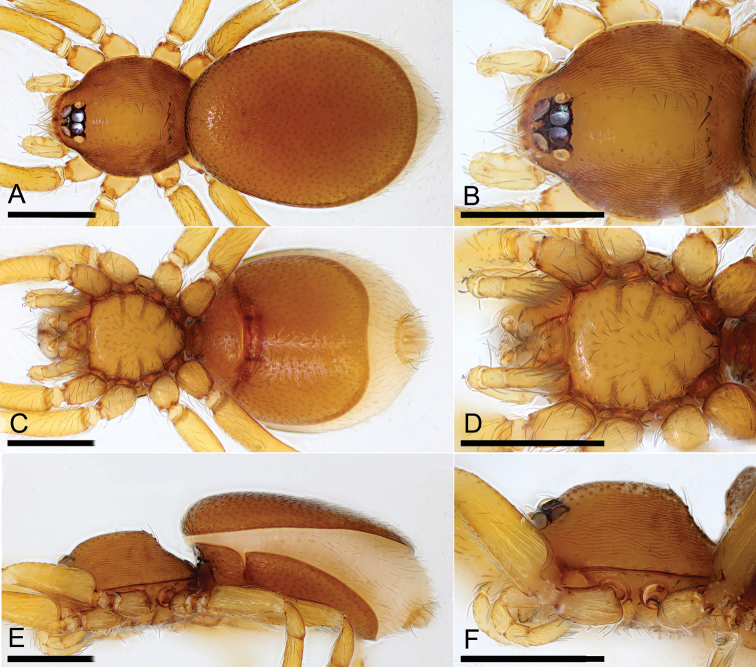
*Opopaea kanpetlet* sp. nov., female (IZCAS Ar-25099) **A, C, E** habitus, dorsal, ventral and lateral views **B, D, F** prosoma, dorsal, ventral and lateral views. Scale bars: 0.4 mm.

Figures 1 , 2 , 3 , 7A–C

###### Type material.

***Holotype***: ♂ (IZCAS Ar-25098), sifting leaf litter, Myanmar, Chin, Roadside between Kanpetlet and Nat Ma Taung National Park, 003, 21°13.325'N, 93°55.739'E, 2942 m, 30.IV.2017, Wu J & Chen Z. ***Paratypes***: 2♀ (IZCAS Ar-25099, 25100), same data as holotype; 1♂ (IZCAS Ar-25101), 3♀ (IZCAS Ar-25102, 25103, 25104), sifting leaf litter, Myanmar, Chin, near 16.5 km of the roadside between Kanpetlet and Nat Ma Taung National Park, 002, 21°13.195'N, 93°16.125'E, 2789 m, 30.IV.2017, Wu J & Chen Z.

###### Etymology.

The specific name is a noun in apposition taken from the type locality.

###### Diagnosis.

The new species is similar to *Opopaea tumida* Tong & Li, 2013, but can be distinguished by the small booklung covers (Fig. 1I), the acute tip of male palpal bulb (Figs 1J–L, 2) and the posterior scutal ridge of the female (Fig. 7A–C). The male of *O. tumida* has large booklung covers, a small apophysis in the retrolateral distal region of the palpal bulb, and the female is lacking the posterior scutal ridge (Tong and Li 2013: figs 8I, 9I, J, 10D).

###### Description.

**Male** (holotype). ***Measurements***: TL: 1.63; CL: 0.68; CW: 0.58; AL: 0.91; AW: 0.69; ALE: 0.08; PME: 0.07; PLE: 0.06; EGW: 0.23; ALE-ALE: 0.04; ALE-PLE: 0.01; PME-PME: 0; PLE–PME: 0; CBL: 0.23; CBW: 0.08; PTL: 0.33; FI: 0.16; FML: 0.13. ***Coloration***: legs yellow, carapace and abdomen scuta yellow brown, abdominal interscutal areas creamy white, booklung covers light yellow, pedipalps reddish brown. ***Habitus*** as in Fig. 1A, C, E. Carapace (Fig. 1B, F): wide oval in dorsal view; sides with longitudinal streaks; median area smooth with rows of setae at lateral edges. ***Eyes*** (Fig. 1B, G): ALE largest, PLE smallest; posterior eye row recurved viewed from above, procurved from front; ALE separated by less than their radius, ALE–PLE separated by less than ALE radius, PME touching throughout most of their length, PLE–PME separated by less than PME radius. Clypeus height about 1.1 times ALE diameter (Fig. 1G). ***Sternum*** (Fig. 1D) longer than wide, fused to carapace; surface smooth; radial furrows present between coxae I-II, II-III, III-IV, with rows of small pits. Endites anteriorly with a small, sharply pointed projection. ***Abdomen***: booklung covers very small, ovoid, without setae. Pedicel tube short, ribbed, with small, dorsolateral triangular extensions, scuto-pedicel region lower than pedicel diameter, with arched scutal ridges, interrupted medially, with curved anterior scutal ridge (Fig. 1H, I). ***Palp*** (Figs 1J–L, 2): femur slightly shorter than half length of patella and submedially attached to patella; patella strongly enlarged, elongate oval; tibia small, rounded; cymbiobulbus shorter than the patella; palpal fenestra small oval and located nearly at tip of cymbiobulbus. Tip of embolus acute triangle.

**Female** (*n* = 5). As in male except as noted. ***Measurements*** (IZCAS Ar-25099): TL: 1.89; CL: 0.70; CW: 0.61; AL: 1.27; AW: 0.75; ALE: 0.08; PME: 0.06; PLE: 0.05; EGW: 0.21; ALE-ALE: 0.03; ALE-PLE: 0.01; PME-PME: 0; PLE-PME: 0. ***Palp*** light yellow. ***Habitus*** as in Fig. 3A, C, E. Endites without projections. ***Copulatory organ*** (Fig. 7A–C): posterior margin of epigastric scutal ridge (asr) smooth, thick posterior scutal ridge (psr) adjacent to asr, small postgynal semicircular depression (pd) between asr and psr; dorsally with nail-like process (nlp) connected to paddle-like sclerite (pls) bearing thin, straight arms.

###### Distribution.

Known only from the type locality.

##### Opopaea zhigangi

Taxon classificationAnimaliaAraneaeOonopidae

Tong & Li sp. nov.

ED803F05-59C8-5E59-A842-67B70EA1C1CF

http://zoobank.org/21168390-94EC-4387-9CA9-3ADF1526DEE7

**Figure 4.**
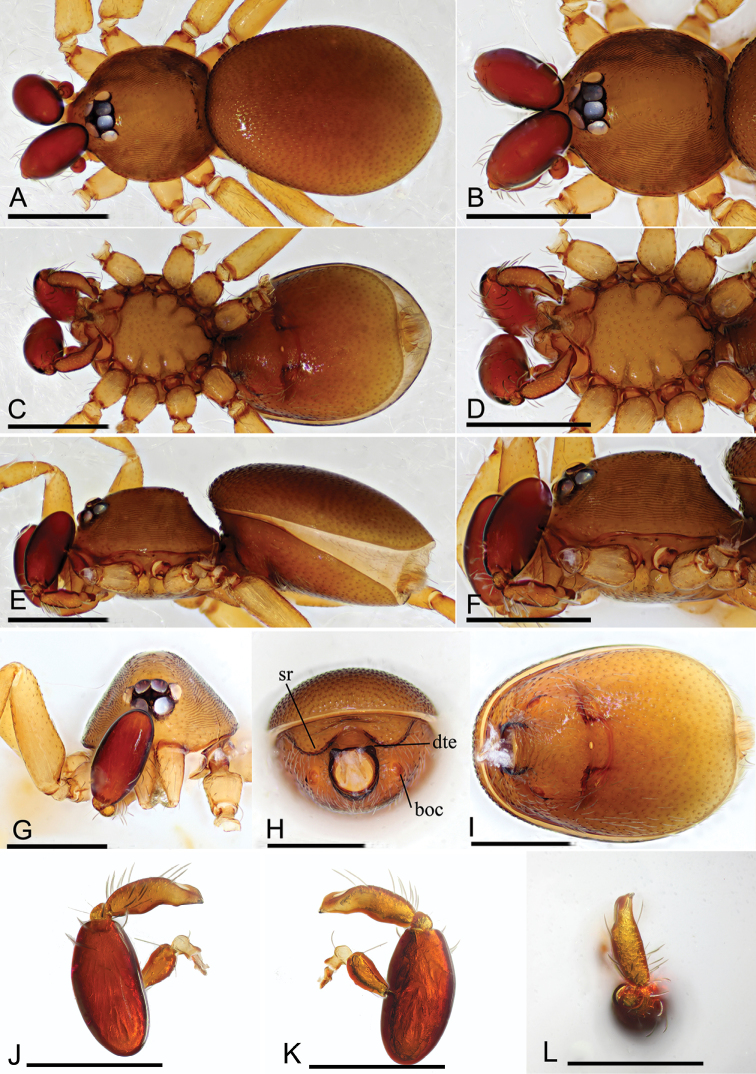
*Opopaea zhigangi* sp. nov., male holotype **A, C, E** habitus, dorsal, ventral and lateral views **B, D, F, G** prosoma, dorsal, ventral, lateral and anterior views **H, I** abdomen, anterior and ventral views **J, K, L** left palp, prolatral, retrolateral and dorsal views. Abbreviations: boc = booklung covers; dte = dorsolateral, triangular extensions; sr = scutal ridge. Scale bars: 0.4 mm.

**Figure 5.**
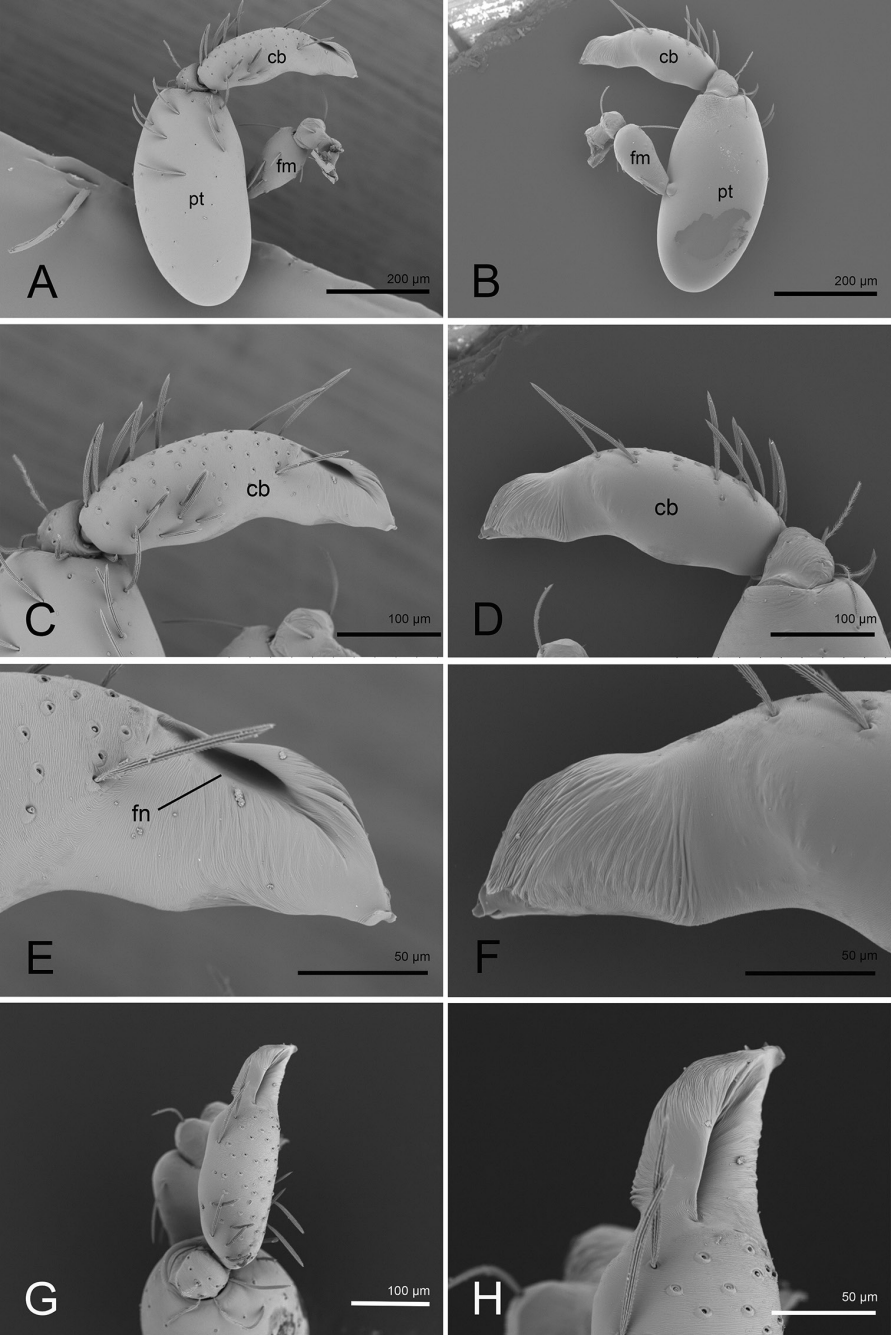
*Opopaea zhigangi* sp. nov., holotype, left male palp, SEM**A, B** prolateral and retrolateral views **C, D, G** cymbiobulbus, prolateral, retrolateral and dorsal views **E, F, H** distal part of cymbiobulbus, prolateral, retrolateral and dorsal views. Abbreviations: cb = cymbiobulbus; fm = femur; fn = fenestra; pt = patella.

**Figure 6.**
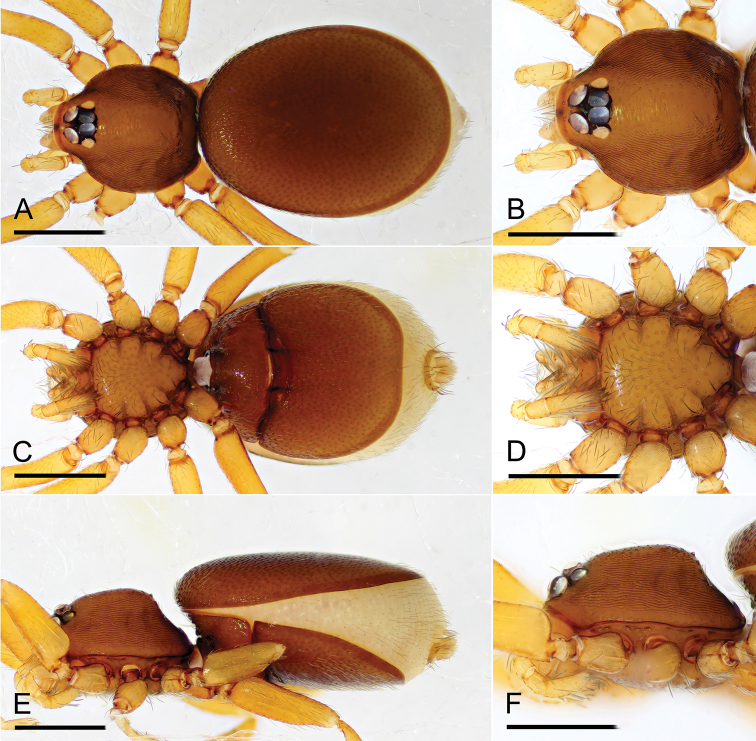
*Opopaea zhigangi* sp. nov., female (IZCAS Ar-25106) **A, C, E** habitus, dorsal, ventral and lateral views **B, D, F** prosoma, dorsal, ventral and lateral views. Scale bars: 0.4 mm.

**Figure 7.**
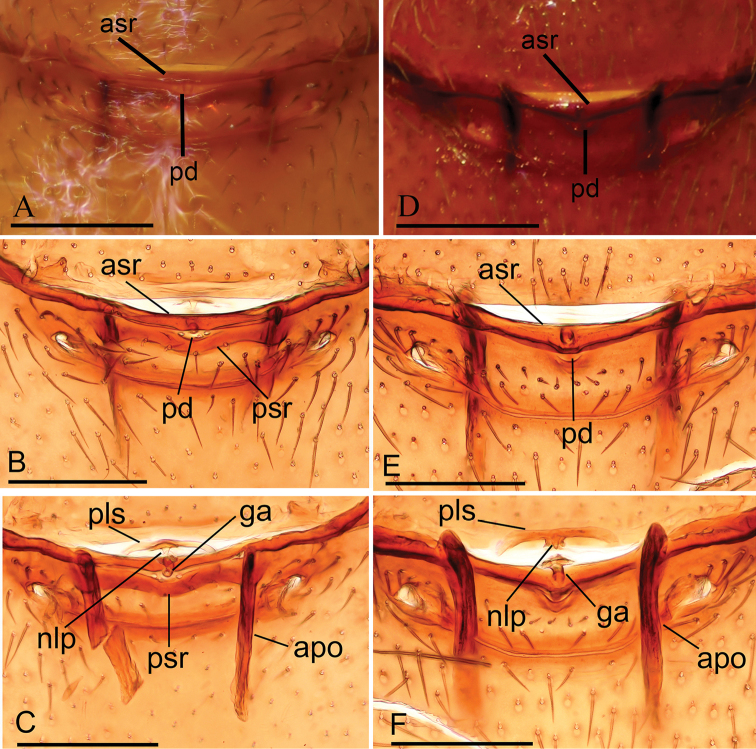
Female copulatory organ **A–C***Opopaea kanpetlet* sp. nov. (IZCAS Ar-25099) **D–F***Opopaea zhigangi* sp. nov. (IZCAS Ar-25106) **A, B, D, E** ventral view **C, F** dorsal view. Abbreviations: apo = apodeme; asr = anterior scutal ridge; ga = globular appendix; nlp = nail-like process; pd = postgynal depression; pls = paddle-like sclerite; psr = posterior scutal ridge. Scale bars: 0.1 mm.

Figures 4 , 5 , 6 , 7D–F

###### Type material.

***Holotype***: ♂ (IZCAS Ar-25105), sifting leaf litter, Myanmar, Chin, near 1.5 km of the roadside between Kanpetlet and Nat Ma Taung National Park, 011–012, 21°13.058'N, 93°59.033'E, 2421 m, 1.V.2017, Wu J & Chen Z. ***Paratype***: 1♀ (IZCAS Ar-25106), same data as holotype.

###### Etymology.

The specific name is after Mr Zhigang Chen, one of the collectors of this species; noun (name) in genitive case.

###### Diagnosis.

The new species is similar to *Opopaea deserticola* Simon, 1892, but can be distinguished by the longer palpal patella (the ratio of width to length about 0.5) and slender cymbiobulbus (the ratio of width to length about 0.35) of male (Figs 4J–L, 5) and very small postgynal depression of female (Fig. 7E). The male of *O. deserticola* has relatively shorter palpal patella (the ratio of width to length about 0.65) and expanded cymbiobulbus (the ratio of width to length about 0.47) and the female has a relatively larger postgynal depression (Platnick and Dupérré 2009: figs 55–66).

###### Description.

**Male** (holotype). ***Measurements***: TL: 1.54; CL: 0.60; CW: 0.58; AL: 0.98; AW: 0.70; ALE: 0.08; PME: 0.07; PLE: 0.07; EGW: 0.22; ALE–ALE: 0.02; ALE–PLE: 0.01; PME–PME: 0; PLE–PME: 0; CBL: 0.31; CBW: 0.11; PTL: 0.41; FI: 0.21; FML: 0.14. ***Coloration***: legs yellow, carapace and abdomen scuta brown, abdominal interscutal areas creamy white, booklung covers reddish, pedipalps reddish brown. ***Habitus*** as in Fig. 4A, C, E. Carapace (Fig. 4B, F): wide oval in dorsal view; sides with longitudinal streaks; median area smooth with some setae at lateral edges. ***Eyes*** (Fig. 4B, G): ALE largest, PLE smallest; posterior eye row recurved viewed from above, procurved from front; ALE almost touching, ALE–PLE separated by less than ALE radius, PME touching throughout most of their length, PLE–PME separated by less than PME radius. Clypeus height about 1.7 times ALE diameter (Fig. 4G). ***Sternum*** (Fig. 4D) longer than wide, fused to carapace; surface smooth; radial furrows present between coxae I–II, II–III, III–IV, with rows of small pits. Endites anteriorly with small, sharply pointed projection. ***Abdomen***: booklung covers very small, reddish brown, ovoid, without setae. Pedicel tube short, ribbed, with small, dorsolateral, triangular extensions, scuto-pedicel region lower than pedicel diameter, with arched scutal ridges, with curved anterior scutal ridge (Fig. 4H, I). ***Palp*** (Figs 4J–L, 5): femur slightly shorter than half length of patella and submedially inserted to patella; patella very large; tibia small, rounded; cymbiobulbus shorter than the patella; fenestra large slit-like, located at nearly tip of cymbiobulbus. Tip of embolus broad triangle.

**Female** (*n* = 1). As in male except as noted. ***Measurements***: TL: 1.84; CL: 0.65; CW: 0.58; AL: 1.18; AW: 0.87; ALE: 0.09; PME: 0.07; PLE: 0.06; EGW: 0.21; ALE-ALE: 0.02; ALE–PLE: 0.01; PME–PME: 0; PLE–PME: 0. ***Habitus*** as in Fig. 6A, C, E. Endites without projections. ***Copulatory organ*** (Fig. 7D–F): postgynal depression (pd) very small; dorsally with nail-like process (nlp) connected to paddle-like sclerite (pls) bearing thick, straight arms.

###### Distribution.

Known only from the type locality.

## Supplementary Material

XML Treatment for Opopaea kanpetlet

XML Treatment for Opopaea zhigangi
